# Reference Values of Maximal Oxygen Uptake for Polish Rowers

**DOI:** 10.2478/hukin-2014-0117

**Published:** 2014-12-30

**Authors:** Andrzej Klusiewicz, Michał Starczewski, Maria Ładyga, Barbara Długołęcka, Wojciech Braksator, Artur Mamcarz, Dariusz Sitkowski

**Affiliations:** 1 Department of Physiology, Institute of Sport, Warsaw, Poland.; 2 Department of Physiology and Biochemistry, The Josef Pilsudski University of Physical Education in Warsaw, Faculty of Physical Education and Sport in Biala Podlaska, Poland.; 3 Department of Cardiology, Hypertension and Internal Diseases, Medical University of Warsaw, Warsaw, Poland.; 4 III Department of Cardiology and Internal Diseases, Medical University of Warsaw, Warsaw, Poland.

**Keywords:** Polish rowers, VO2max, reference values

## Abstract

The aim of this study was to characterize changes in maximal oxygen uptake over several years and to elaborate current reference values of this index based on determinations carried out in large and representative groups of top Polish rowers. For this study 81 female and 159 male rowers from the sub-junior to senior categories were recruited from the Polish National Team and its direct backup. All the subjects performed an incremental exercise test on a rowing ergometer. During the test maximal oxygen uptake was measured with the BxB method. The calculated reference values for elite Polish junior and U23 rowers allowed to evaluate the athletes’ fitness level against the respective reference group and may aid the coach in controlling the training process. Mean values of VO2max achieved by members of the top Polish rowing crews who over the last five years competed in the Olympic Games or World Championships were also presented. The results of the research on the “trainability” of the maximal oxygen uptake may lead to a conclusion that the growth rate of the index is larger in case of high-level athletes and that the index (in absolute values) increases significantly between the age of 19–22 years (U23 category).

## Introduction

For several dozen years maximal oxygen uptake (VO2max) has been regarded as the primary physiological index used for the assessment of the organism’s cardio-pulmonary fitness and predisposition towards success in endurance sports (Hue et al., 2000; Klusiewicz et al., 1999; Legaz-Arresea et al., 2007; Losnegard and Hallén, 2014; Millet et al., 2003; Suriano and Bishop, 2010; Yagüe et al., 2013). Indeed, a strong relationship between the place taken in international regattas and VO2max values achieved by the athletes has been demonstrated (Secher et al., 1982). Currently, based on long-term observations of an outstanding senior rower, a medal winner at seven Olympic Games and World Championships, a correlation has been suggested between competition results and the capacity to maximally uptake high amounts of oxygen (about 6.0 l/min) and the peak power attained in the incremental exercise test on a rowing ergometer (Lacour et al., 2009). The athlete’s capability to withstand heavy training workload until the age of 32 was an additional factor in this case. Another study (Mikulic, 2011) showed that within a five-year observation period, starting from the age of 16 years, the world–class rowers demonstrated a systematic and significant increase (26%) in the maximal oxygen uptake up to the level of about 6.6 l/min (i.e. 70 ml/kg/min).

We attempted to describe multi-year changes in maximal oxygen uptake, and we elaborated up-to-date reference values of this index based on studies of large and representative groups of Polish elite rowers.

## Material and Methods

The cross-sectional studies have been conducted over the last four years on numerous groups of female (n=81) and male (n=159) rowers from the sub-junior to senior categories of the Polish National Team and its direct back-up. Approval from the Research Ethics Committee of the Institute of Sport in Warsaw had been granted before the commencement of the tests and informed written consent to participate in the study had been obtained from all subjects.

All the participants performed an incremental exercise test until exhaustion on the Concept II rowing ergometer at the workloads shown in Table 1. The test consisted of three-minute exercise bouts separated by 30 s rest periods (Klusiewicz, 2005).

During the exercise test, the HR was continuously monitored with the use of a Polar S610i recorder (Polar Electro Oy, Finland). The respiratory exchange indices were measured with the BxB method using the MetaLyser 3B and MetaMax 3B devices (Cortex, Germany). Three minutes after the incremental exercise bouts until exhaustion, blood samples were collected for determination of the lactate concentration with the use of the Super GL 2 (Dr Müller, Germany) devices.

Maximal oxygen uptake (VO_2max_) was defined as the highest amount of oxygen consumed by the athlete’s organism during one minute of the test. The maximal intensity exercise necessary for estimation of VO_2max_ was defined by the following criteria: the VO_2_ plateauing with increasing workload, the post-exercise blood lactate concentration >8 mmol/l, the Respiratory Exchange Ratio (RER) >1.1 and the attainment of the age-adjusted maximal heart rate expressed as HRmax = 220 – age of the subject. If at least two of the above criteria were met during the exercise, the attained effort and oxygen uptake were regarded as maximal.

For statistical analysis of the results means (x) and standard deviations (SD) of the examined parameters (x) were calculated. The Shapiro-Wilk test was used to check if the distribution of examined variables was normal. To compare the results, one-way analysis of variance (ANOVA) was used. For detailed comparisons (between groups) the post-hoc Tukey’s test for unequal samples was utilised. The level of statistical significance was set at p<0.05. For all the calculations and statistical analyses of the results the Statistica v.8 (StatSoft) software was used.

## Results

The reference values for maximal oxygen uptake (Table 2) are based on the results obtained during the exercise test in numerous groups of female and male rowers (sub-juniors, juniors, and U23) recruited from the Polish athletes of a different sports level.

The analysis of changes in the maximal oxygen uptake with age (expressed in l/min) showed significantly higher scores of the factor than the starting rate (age 15–15.9) only in senior female rowers (age 20–20.9 and 21–22). In male rowers substantial improvement was observed at the age of 19–19.9 and 21–22. Interestingly, maximal oxygen uptake in the context of body mass did not show significant changes within the age range from 15 to 22 neither in female nor in male athletes.

## Discussion

The main goal of the present study was to quantify maximal oxygen uptake in rowers from different age categories and competition levels. The calculated reference values for elite Polish junior and U23 rowers allowed to evaluate the athletes’ fitness level against the respective reference group and may aid the coach in controlling the training process (Table 2). Moreover, the data obtained from studies carried out over the last four years in elite Polish crews, medallists at the Olympic Games and World Championships, indicate that oxygen uptake of top female and male rowers markedly exceeds 4.0 and 6.0 l/min, respectively. In lightweight athletes this uptake, expressed in relative units, exceeded 70 ml/kg/min (Table 3). Other authors demonstrated that a male rower, a winner of numerous medals at the Olympic Games and World Championships, attained VO2max of 6.26±0.05 l/min (Lacour et al., 2009), whereas the mean value obtained for world elite quadruple scull rowers was 6.62 l/min (the highest individual value was 6.97 l/min) (Mikulic, 2011). As indicated by a number of reports from studies of female and male rowers (Klusiewicz et al., 1999; Lacour et al., 2009; Mejuto et al., 2012; Secher et al., 1982) maximal oxygen uptake is of high diagnostic value when it is expressed in absolute values (l/min) or – in case of lightweight athletes whose body mass is strictly regulated and stable – in relative values (ml/kg/min).

**Figure 1:**
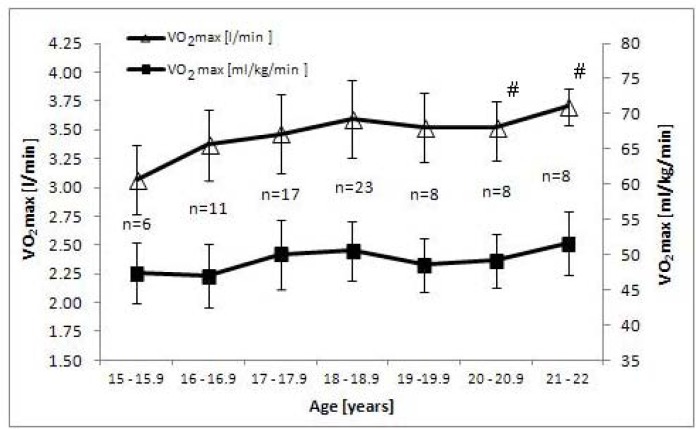
Maximal oxygen uptake (VO_2max_) in the examined female rowers from various age groups (n=81). # - significantly different from the 15-years old level (p<0.05)

In practical terms, it was important to evaluate a trend in the development of maximal oxygen uptake to get insight into the degree of improvement of the examined index in rowers. Such an analysis was based on studies carried out in Polish rowers and the results are plotted in Figures 1 (for females) and 2 (for males). Cross-sectional data available for these athletes demonstrate that the highest significant increases in absolute values of VO2max were recorded for 20-22-year-old females and the 19-19.9 and 21-22-year-old males. Analysis of the data expressed in relative values hardly changed the trend described above for female rowers, while a marked decrease of the peak oxygen uptake was recorded in male rowers older than 19 years (statistically non significant). Taking into account the dynamics of annual changes, longitudinal studies of successful Croatian quadruple scull rowers demonstrated the highest improvement (by 6.8%) of maximal oxygen uptake expressed in absolute values at the age of 19.2–20.2 years. Stabilization of the values of this index was detected at the age of about 20 years (Mikulic, 2011). In the Croatian rowers, the total “trainability” of VO_2max_ over the five-year period equalled to 25.9%, whereas during the seven-year observation of Polish rowers it amounted to 22.0 and 11.7% in females and males, respectively.

Other authors reported that in top rowers VO2max peaked at the age of 23 years and then slightly increased until 28 years of age (Messonier et al., 1998). A similar tendency was observed in elite runners above the age of 20–22 years (Legaz Arrese et al., 2005). Likewise, Lacour et al. (2009) based on the published data described the age-related decrease (beginning from 20–25 years) in physical fitness and VO2max in long-distance runners and swimmers. Murase et al. (1981) in their studies observed in the elite junior runners an increase in VO2max from 3.54 l/min (65.4 ml/kg/min) to 4.49 l/min (75.5 ml/kg/min) between the age of 14.8 and 18.8. Similarly, Rusko (1992) noticed that maximum oxygen uptake values improved with training in cross country skiers from approximately 55–60 to 75–80 ml/kg/min between the age of 15 and 25. On the other hand Ingjer (1992) observed a development in VO2max in ml/kg/min only till 15–16th year of age, however, maximal oxygen uptake (expressed in l/min) in the tested leading Scandinavian skiers continued to improve until the age of 20. In this context, the results reported by Lacour et al. (2009) for an outstanding senior rower whose maximal oxygen uptake continued elevating until 32 years of age are quite exceptional.

The results of the research on the “trainability” of maximal oxygen uptake may lead to a conclusion that the growth rate of this index is larger in case of high-level athletes and that it (in absolute values) increases significantly between the age of 19–22 years (U23 category).

## Figures and Tables

**Figure 2:**
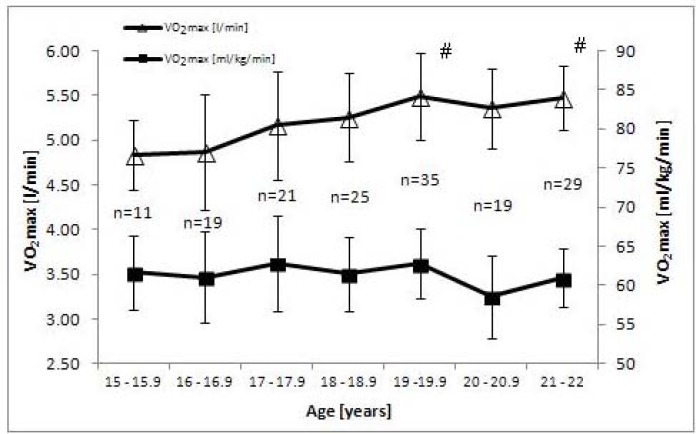
Maximal oxygen uptake (VO_2max_) in the examined male rowers from various age groups (n=159). # - significantly different from the 15-years old level (p<0.05)

**Table 1:** Workloads applied during the incremental exercise test in female (n=81) and male (n=159) rowers

Workload/Power [W]	I	II	III	IV	V	VI	VII
Stroke rate [1/min]	16	18	20	22	24	26	28
Female rowers/Category							
Sub-juniors	80	120	160	200	240	280	320
Juniors	100	140	180	220	260	300	340
U23	120	160	200	240	280	320	360
Seniors	120	160	200	240	280	320	360
Lightweight	80	120	160	200	240	280	320
Male rowers/Category							
Sub-juniors	120	160	200	240	280	320	360
Juniors	150	190	230	270	310	350	390
U23	170	220	270	320	370	420	470
Seniors	220	270	320	370	420	470	520
Lightweight juniors	120	160	200	240	280	320	360
Lightweight U23	120	170	220	270	320	370	420

**Table 2:** Reference values of maximal oxygen uptake (VO_2max_) defined for the examined categories of female (n=81) and male (n=159) rowers

	VO_2max_ [l/min]			VO_2max_ [ml/kg/min]		

Assessment Category	Low	Average	High	Low	Average	High
Females						

FSJ (n=9)	3.0 ≤	3.0 – 3.3	≥ 3.3	45.1 ≤	45.1 – 49.1	≥ 49.1
FJ (n=39)	3.3 ≤	3.3 – 3.6	≥ 3.6	47.0 ≤	47.0 – 51.5	≥ 51.5
FY (n=24)	3.5 ≤	3.5 – 3.8	≥ 3.8	49.5 ≤	49.5 – 54.9	≥ 54.9
FYLW (n=9)	3.2 ≤	3.2 – 3.5	≥ 3.5	55.6 ≤	55.6 – 58.3	≥ 58.3

Males						

MSJ (n=11)	4.6 ≤	4.6 – 5.0	≥ 5.0	59.3 ≤	59.3 – 64.1	≥ 64.1
MJ (n=52)	4.7 ≤	4.7 – 5.3	≥5.3	58.8 ≤	58.8 – 64.5	≥ 64.5
MY (n=58)	5.3 ≤	5.3 – 5.8	≥ 5.8	59.8 ≤	59.8 – 64.8	≥ 64.8
MJLW (n=10)	4.3 ≤	4.3 – 4.8	≥ 4.8	62.0 ≤	62.0 – 67.4	≥ 67.4
MYLW (n=32)	4.7 ≤	4.7 – 5.1	≥ 5.1	65.0 ≤	65.0 – 70.0	≥ 70.0

Parameter level: low < (x − 0.5 SD), average = (x ± 0.5 SD), high > (x + 0.5 SD) FSJ – female sub-juniors, FJ – female juniors, FY – female U23, FYLW – female U23 lightweight MSJ – male sub-juniors, MJ – male juniors, MY – male U23, MJLW – male junior lightweight, MYLW – male U23 lightweight

**Table 3:** The highest mean values of maximal oxygen uptakes (VO_2max_) attained by the top Polish rowing crews who over the last five years competed in the Olympic Games (O.G.) and World Championships (W.Ch.)

Crew	Regatta	Place taken	VO_2max_	
			[l/min]	[ml/kg/min]
	O.G. 2012	III		
2xKA	W.Ch. 2010	III	4.45±0.11	63.3±1.8

	O.G. 2008	I		
4xMA	W.Ch. 2009	I	6.08±0.65	64.9±6.1

	O.G. 2008	II	5.24±0.48	70.7±7.1
4-ML^*^	W.Ch. 2009	III	5.31±0.50	71.9±7.3

2xKA – a women double scull, 4xMA – a men quadruple scull, 4-ML – a men lightweight coxless four

* different members of the crew in 2008 and 2009
